# Impact of facilitating continued accessibility to cancer care during COVID-19 lockdown on perceived wellbeing of cancer patients at a rural cancer center in Rwanda

**DOI:** 10.1371/journal.pgph.0001534

**Published:** 2023-02-27

**Authors:** Anne Niyigena, Vincent K. Cubaka, Pacifique Uwamahoro, Robert Gatsinzi Mutsinzi, Benigne Uwizeye, Blandine Mukamasabo, Cyprien Shyirambere, Bosco Jean Bigirimana, Joel Mubiligi, Dale A. Barnhart

**Affiliations:** 1 Partners In Health, Kigali, Rwanda; 2 Department of Global Health and Social Medicine, Harvard Medical School, Boston, Massachusetts, United States of America; University of Embu, KENYA

## Abstract

During the COVID-19 pandemic in Rwanda, Partners In Health Inshuti Mu Buzima collaborated with the Butaro Cancer Center of Excellence (BCCOE) to mitigate disruptions to cancer care by providing patients with free transportation to treatment sites and medication delivery at patients’ local health facilities. We assessed the relationship between facilitated access to care and self-reported wellbeing outcomes. This cross-sectional telephone survey included cancer patients enrolled at BCCOE in March 2020. We used linear regression to compare six dimensions of quality of life (EORTC QLQ-C30), depression (PHQ-9), anxiety (GAD-7), and financial toxicity (COST) among patients who did and did not receive facilitated access to care. We also assessed access to cancer care and whether patient wellbeing and its association with facilitated access to care differed by socioeconomic status. Of 214 respondents, 34.6% received facilitated access to care. Facilitated patients were more likely to have breast cancer and be on chemotherapy. Facilitation was significantly associated with more frequent in-person clinical encounters, improved perceived quality of cancer care, and reduced transportation-related barriers. Facilitated patients had significantly better global health status (β = 9.14, 95% CI: 2.3, 16.0, p <0.01) and less financial toxicity (β = 2.62, 95% CI: 0.2,5.0, p = 0.03). However, over half of patients reported missing or delaying appointment. Patient wellbeing was low overall and differed by patient socioeconomic status, with poor patients consistently showing worse outcomes. Socioeconomic status did not modify the association between facilitated access to care and wellbeing indicators. Further, facilitation did not lead to equitable wellbeing outcomes between richer and poorer patients. Facilitated access to care during COVID-19 pandemic was associated with some improvements in access to cancer care and patient wellbeing. However, cancer patients still experienced substantial disruptions to care and reported low overall levels of wellbeing, with socioeconomic disparities persisting despite facilitated access to care. Implementing more robust, equity-minded facilitation and better patient outreach programs during health emergencies may promote better care and strengthen patient care overall and effect better patients’ outcomes.

## Introduction

COVID-19 drastically disrupted cancer care in high income countries. In an effort to efficiently use healthcare infrastructure during the peak waves of the COVID-19 pandemic, guidelines for cancer patient management recommended delay or cancelation of non-urgent cancer care services including referrals, surgery, drug treatment, follow-up, and screening [1–3]. However, due to the time-sensitive nature of cancer treatment, these delays in care provision are expected to result in meaningful increases in patient morbidity and mortality [4, 5].

In low- and middle-income countries (LMICs), where cancer care infrastructure was already constrained before the pandemic, the impact of COVID-19 on cancer care is expected to be even worse [3, 6, 7]. Cancer patients in Africa and Southeast Asia experienced social and economic barriers to accessing health facilities, and even patients with urgent needs had their cancer treatments disrupted [7–9]. While some high-income countries (HICs) alleviated similar barriers through telemedicine [10–12], poor connectivity and limited access to technology impeded the adoption of telemedicine in LMICs during the pandemic [13–15].

Just as the COVID-19 pandemic has increased disparities in access to cancer care between high- and low-income countries, within-country disparities in access to cancer care are also increasing [16, 17]. In both high- and low-income countries, patients with lower socioeconomic status experience greater worry about affording long-term expenses related to cancer treatment and follow up [18]. Because both financial and psychologic wellbeing are associated with patients’ compliance to their cancer management plan, mitigating the impact of the COVID-19 pandemic on financial and psychologic health may be one way to enhance survival among cancer patients and promote equitable outcomes [19–21].

While many cancer care institutions have adapted interventions to mitigate care interruptions, many of these interventions were targeted at facility- or service delivery-levels and hence their evaluations did not measure the impact of such mitigation strategies on patient’s level [8, 22–24]. To our knowledge, no previous study has documented the impact of care continuation interventions on the quality of life, mental health, and socioeconomic wellbeing of cancer patients. Our study describes the wellbeing of cancer patients who were enrolled in cancer care at the Butaro Cancer Center of Excellence in Rwanda at the start of the COVID-19 pandemic and assesses the association between receipt of facilitated access to care during COVID-19 lockdowns and patients’ wellbeing, including overall quality of life, mental health, and socioeconomic outcomes. We also evaluate whether the association between facilitated access to care and wellbeing differed by patients’ baseline socioeconomic status.

## Methods

### Study setting

Cancer care in Rwanda is mainly delivered at two cancer centers: the Butaro Cancer Center of Excellence (BCCOE), which was founded in 2012 and is located in the most rural district of Rwanda, and the Rwanda Cancer Center (RCC), which was founded in 2019 and is located in the capital city, Kigali. Currently, most cancer patients are managed at BCCOE whereas RCC primarily provides radiation therapy and chemotherapy for outpatients. Although BCCOE is largely government supported, since its founding it has also been supported by Partners in Health/ Inshuti Mu Buzima (PIH/IMB), the Rwandan branch of an international non-governmental organization that accompanies the Rwandan Ministry of Health in strengthening Rwanda’s health system. At BCCOE, medications, cost of treatment, transportation to cancer health facilities are substantially subsidized for poorer patients in the first and second *ubudehe* categories. The *ubudehe* system is a 4-tier socio-economic classification system used by the government to identify households that should benefit from social protection programs, where category one roughly corresponds to living in acute poverty and category two corresponds to living a substance lifestyle with no surplus income [25]. Patients come from different rural and urban locations across the country to seek care and treatment at BCCOE and breast cancer stands as the most common types of cancer treated and managed at this rural center.

The first case of COVID-19 was reported in Rwanda on March 22^nd^, 2020 and was followed by an abrupt total lockdown until May 31^st^, 2020 with subsequent partial lockdowns. Although routine health care activities did not officially close during lockdowns, the cessation of other key services such as public transportation and household livelihood activities meaningfully disrupted access to and delivery of health care. During this time, Rwanda Ministry of Health (RMoH) collaborated with its key partners, including PIH/IMB, to promote continuity of cancer care services for patients in Rwanda and East Africa. These activities included a) expansion of free transportation to all cancer patients managed at BCCOE residing in Rwanda, where any patient who needed a consultation or infusion chemotherapy were eligible for round-trip transportation from their home to BCCOE regardless of their *ubudehe* category; and b) aerial or vehicle-based delivery of oral chemotherapy medication to local health centers of patients residing in remote areas. Over 40% of cancer patients managed at BCCOE received some sort of facilitated access to care from PIH/IMB during COVID-19 lockdowns in Rwanda. More details on the role of PIH/IMB in the decentralization of cancer care during COVID-19 lockdowns has been described elsewhere [24].

### Study design and population

We conducted a cross-sectional telephone survey among adult cancer patients who received cancer care at BCCOE and compared care provision, overall quality of life, mental health, and socioeconomic outcomes among patients who did and did not receive facilitated access to care during COVID-19 pandemic. Patients were eligible for inclusion if they were enrolled in cancer care at BCCOE by March 22^nd^ 2020, resided in Rwanda, had a telephone number recorded in the Electronic Medical Record (EMR), and were still alive at the time of data collection in August of 2021. We excluded patients under 18 years old, patients who initiated cancer treatment for the first time during the COVID-19 pandemic, patients without a confirmed diagnosis of cancer at the start of the pandemic, and patients who were confirmed dead by the time of data collection.

### Sample selection

We performed stratified simple random sampling to select study participants who did and did not receive facilitated access to care. Patients who received facilitated access to care were sampled from a list of beneficiaries, which was provided by the PIH/IMB’s oncology program that coordinated the support activities during the pandemic. Non-facilitated patients were drawn from EMR system managed at BCCOE. The non-supported were active cancer patients recorded in EMR whom we did not find on the oncology program list of those who have been supported during COVID-19 pandemic.

**Fig 1:**
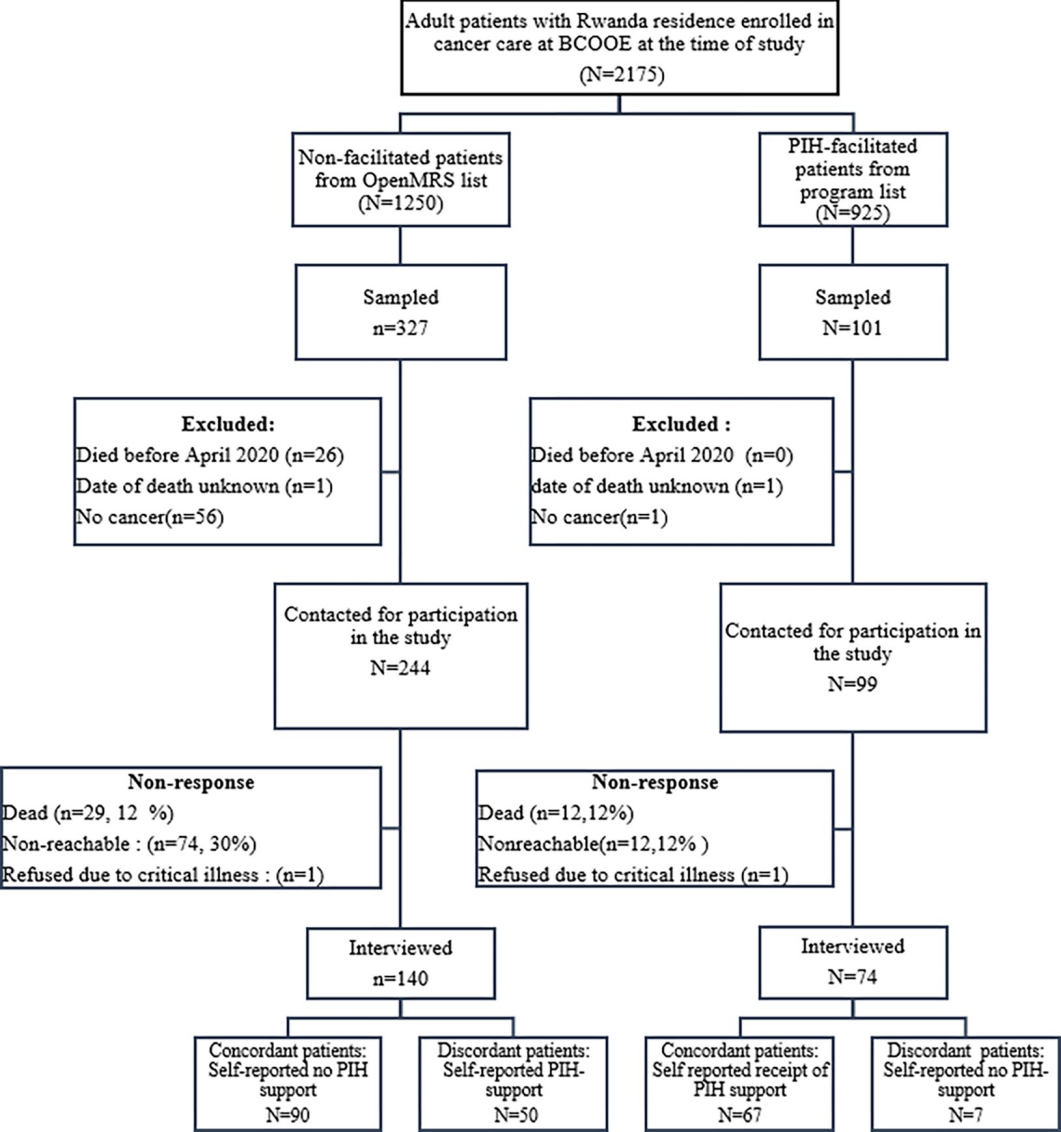
Flowchart of study participants.

We powered our study to detect a 10-point increase in quality of life using the European Organization for the Research and Treatment of Cancer Quality of Life (EORTC QLQ-C30) scale, which would correspond to effect size previously suggested as clinically meaningful if care needs of the newly diagnosed cancer patients are met [26]. Assuming a 5% type 1 error rate; mean EORTC QLQ-C30 of 49.5 in the non-facilitated group and standard deviation of 25.4 which align with mean score and standard deviation of EORTC QLQ-C30 reported by studies from similar settings; and non-equal number of facilitated and non-facilitated group members, with 40% of study participants being sampled from the list of beneficiaries of facilitated transport to reflect the overall proportion of cancer patients having received facilitated access to care from PIH/IMB; we estimated that we would require a total sample size of 214 to detect a 10-percentage point difference in EORTC QLQ-C30 with at least 80% power [27, 28]. This sample of 214 was expected to include 86 participants for the facilitated group and 126 for the non-facilitated group. Since our sampling frames lacked up-to-date information on the current status of the patients, we anticipated a high non-response rate due to death or loss to follow-up. Thus, we conducted random sampling with replacement on both frames until interviewed a total 214 participants. Our final sample of patients interviewed included 74 who received facilitated access to care and 140 who did not receive facilitation (Fig 1).

### Data collection

We conducted telephone-based data collection between September 6^th^and October 6^th^, 2021. The delay between the start of facilitation data collection reflects the fact that the primary goal of the program was to support clinical care, and the idea to conduct an evaluation occurred several months after facilitation began. Patients were recruited via phone calls and provided with information about the study. Those who were interested provided their voluntary informed verbal consent and set up an appointment for data collection. Data collectors confirmed that participants consented to the study before starting data collection. We made three phone-call attempts on three different days before a selected patient was considered to be non-responsive. For telephone numbers that were reachable but which were linked to patients who had died, we briefly interviewed the person who answered the call to ascertain the date of death and the cause of death. Data were collected via a 35-minute phone interview by research staff who were not cancer care providers. Data were stored on a password-protected REDCap database, a data management application first developed by Vanderbilt University and hosted on a Rwanda-based server [29, 30].

### Ethic statement

This study received approval from the Partners In Health/Inshuti Mu Buzima (PIH/IMB) Research Committee (IMBRC) and was conducted under an umbrella protocol for oncology research which was approved by the Rwandan National Ethics Committee (No. 804/RNEC/2021). Voluntary informed verbal consent was obtained from participants before phone-based data collection.

### Conceptual framework

**Fig 2:**
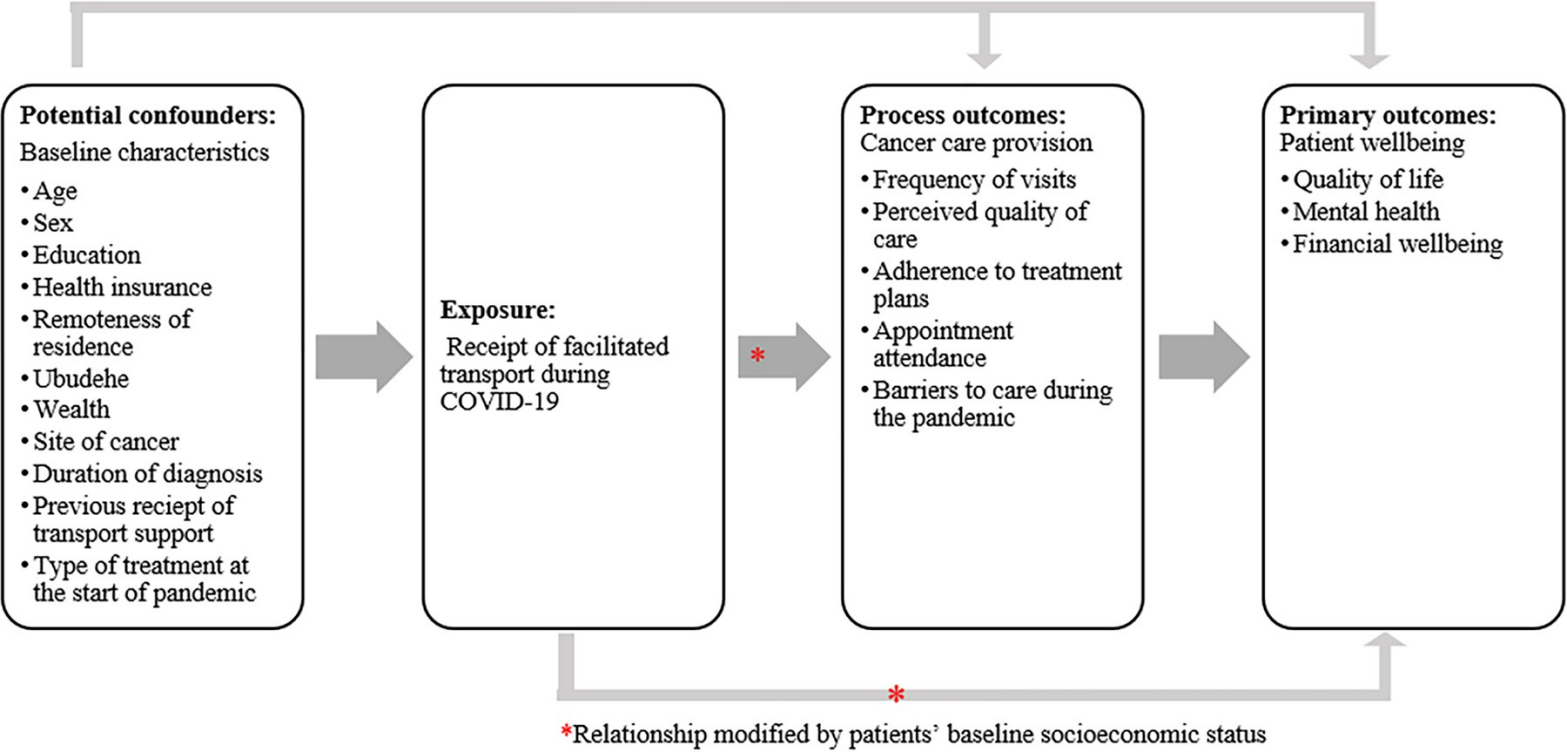
Conceptual framework describing the relationship between patient characteristics, receipt of facilitated transport, cancer care provision, and patient wellbeing.

To understand the relationship between receiving facilitated access to care and improved patient wellbeing, we developed a conceptual framework that considers four types of variables: baseline characteristics, receipt of facilitated access to care, cancer care provision, and patient wellbeing. Baseline characteristics included patients’ demographic and clinical characteristics. Although these characteristics were collected at the same time as outcome ascertainment, they reflected characteristics that are unlikely to change meaningfully over time and were therefore assumed to reflect patient status at the start of the pandemic. These characteristics could have impacted patients’ likelihood of receiving facilitated access to care during the pandemic as well as subsequent patient outcomes and were therefore considered to be potential confounders. Receiving facilitated access to care was our primary exposure of interest and was assumed to depend on patient characteristics. Cancer care provision outcomes were selected based on the literature and reflected changes in the provision of care following the start of the COVID-19 pandemic. These process outcomes were conceived as helping to explain the ways in which access to facilitated access to care could have improved patient wellbeing. Our primary outcome was patient wellbeing, which was measured according to various dimensions, including quality of life, mental health, and financial wellbeing. Because part of the goal of this intervention was to promote equitable access to health care for the most vulnerable patients, we also wanted to assess whether the association between receipt of facilitated access to care and patient wellbeing differed by patients’ baseline socioeconomic status. The relationship between these four types of variables is shown in Fig 2.

### Covariates: Baseline characteristics

We collected demographic characteristics including continuous age, gender, education, health insurance status, remoteness of residence classified as urban versus rural, and *ubudehe* category. We reported on patient wealth using a simplified 15-item tool on household assets [31]. This wealth index tool was developed using data from the Rwanda’s Demographic Health Survey [32] and can be used to categorize households into nationally representative wealth quintiles. For the purpose of our analysis, we collapsed the wealth quintiles into 3 categories: quintiles 1–3 were classified as poor-middle, quintile 4 was labelled the richer, and quintile 5 as the richest. We also assessed clinical characteristics including cancer site, which was dichotomized as breast cancer and non-breast cancer; duration of cancer diagnosis in years; means of transport to cancer care before the pandemic, which was categorized as PIH/IMB-provided transport, public transport, private transport, and by foot; and type of treatment at the start of COVID-19 pandemic, which was categorized as chemotherapy, non-chemotherapy, or no treatment. We dichotomized types of cancer into breast and non-breast cancer types since breast cancer patients represent the majority of patients at BCCOE and hence were overrepresented among the patients who needed to continue treatment at BCCOE during the pandemic. The non-breast cancer group included: cervical cancer, leukemia, colon, gastro-intestine, Kaposi sarcoma and multiple myeloma.

### Primary predictor: Receipt of facilitated care

Respondents who were sampled from the PIH/IMB oncology program’s list of beneficiaries, which included both patients who received transport to treatment sites by PIH/IMB’s vehicles or receiving medication delivery between Mach 22, 2020 up to the time of data collection, were defined as having received facilitated access to care. Patients who were sampled from EMR system managed at BCCOE and not found on the list of beneficiaries were considered to belong to the non-facilitated group. Our primary analyses used this list-based status to define patient’s exposure status. However, to confirm this classification, during data collection we asked patients to self-report whether they actually received support from PIH/IMB to continue care during COVID-19 pandemic. In general, we believe the list-based status to be a more reliable way to classify whether or not patients received facilitated access to transportation because PIH/IMB provides many types of social support and it is possible that patients who self-reported receipt of support may be reporting on social support other than facilitated access to care during the COVID-19 pandemic. However, as a sensitivity analysis, we conducted two additional sets of analyses to explore the impact of discrepancies between self-reported facilitated access to care and lists-based status on our findings. The first set of sensitivity analysis considered self-reported receipt of facilitated access to care as the primary exposure and the second set of sensitivity analyses was restricted to patients whose list-based and self-reported exposures status were concordant.

### Process outcomes: Cancer care provision

We assessed cancer care provision using self-reported data on whether patients missed or delayed appointments due to COVID-19-related circumstances during the pandemic, the number of in-person clinical visits since the start of the pandemic until the time of data collection (17 months); access to teleconsultation, defined as receiving a telephone or digital call from the patients’ usual oncology health provider to discuss their health during COVID-19 pandemic, perceived changes in the quality of cancer care before and during COVID-10 pandemic which was assessed using a 5-points Likert scale ranging from a much worse, a little worse, about the same, better, and much better; change of treatment plan defined as receiving a different treatment because the usual treatment was not available due to COVID-19 pandemic; care support in the household and barriers to care. Barriers to care experienced during COVID-19 pandemic assessed included financial barriers, family issues, lack of transportation, comorbidities, logistical issues to accessing transportation, worsened cancer disease, and COVID-19 infection. Logistical issues to accessing transportation included barriers to accessing transportation when transport means were available, which included difficulties of reaching pick-up points, affordability, missing transport or walking long distance to reach transport pick-up points. Perceived quality of cancer care during COVID-19 pandemic compared to pre-pandemic care was collapsed into three categories: worse, about the same and better.

### Primary outcomes: Patient wellbeing

*Quality of life* was assessed using the European Organization for the Research and Treatment of Cancer Quality of Life (EORTC QOL-C30) questionnaire, a 30-item health-related quality of life (HRQL) tool which is widely used in cancer research [33]. This tool is designed to evaluate cancer patients on important domains of functioning such as global health status; five functional scales, including physical, emotional, role, cognitive, and social functioning; and common cancer symptoms including fatigue, nausea/vomiting, pain, dyspnea, appetite loss, constipations, diarrhea, and financial difficulties. To assess quality of life, we considered the global health status scale and the five functional scales. All domains of EORTC QLQ-C30 are scored such that scores range from 0 to 100. Higher scores represent better functioning on the global health status and functioning domains [34]. The permission to use the EORCT QOL-C30 questionnaire for academic purposes was obtained from the EORTC QOL-C30 (Request ID: 68934).

*Mental Health* was assessed using the patient health questionnaire (PHQ-9), a 9-item questionnaire on depression symptoms over the past two weeks, and the general anxiety disorder questionnaire (GAD-7), a 7-item tool assessing symptoms of general anxiety over the past two weeks. Scores for the PHQ-9 range between 0 and 27 with high scores corresponding to more severe depression [35, 36]. Scores for the GAD-7 range between 0 and 21 with high scores corresponding to more severe anxiety [37].

To measure *financial wellbeing*, we used a validated COmprehensive Score for financial Toxicity (COST) questionnaire [38]. This 11-item tool has scores ranging between 0 and 44, where higher scores indicate higher financial wellbeing. We additionally used the single-item financial difficulties domain from the EORTC QOL-C30. As with the other domains of the EORTC QOL-C30, the financial difficulties domain is scaled from 0 to 100; however, a higher score corresponds to worse financial difficulties [34].

For all outcomes, (EORTC QLQ-C30, PHQ-9, GAD-7, and COST-FACIT) we followed forward and backward translation procedure recommended by the EORTC translation group [33], to translate tools from English to the local language of Kinyarwanda. Two study team members independently performed the first round of forward translation from English to Kinyarwanda. Both forward translations were reconciled by a third study team member into a single Kinyarwanda version which was back translated into English by a fourth and fifth study team members, working independently from one another. The original English questionnaire, the two forward-translated Kinyarwanda documents, the two back- translated English documents and the reconciled Kinyarwanda documents were scrutinized by the study team to produce a final Kinyarwanda questionnaire, which was administered to the study participants.

### Data analysis

We described baseline characteristics of study participants and cancer care provision outcomes using descriptive and summary statistics, including frequencies and percentages for categorical data and means and standard deviations for continuous outcome data. Variables were described for the overall population and separately by receipt of facilitated access to care. We used Fisher’s Exact tests, chi-square tests or t-tests to assess bivariate associations between receipt of facilitated access to care and patients’ baseline characteristics and process outcomes variables.

For each of our four main outcome variables, we reported means and standard deviations for the overall study population and separately by receipt of facilitated access to care. We assessed for univariate associations using an independent t-test for continuous scores. For each outcome, we also conducted three linear regressions to assess the association between the continuous scores of the four main outcome variables and the receipt of facilitated access to care. For each outcome, we performed a crude analysis, a minimally adjusted analysis controlling for all baseline characteristics that were associated with receipt of facilitated access to care at a p≤0.20 level, and a fully adjusted analysis that adjusted for all baseline characteristics. We reported beta coefficients, 95% confidence intervals and p-values for each model.

As a secondary analysis, we assessed associations between patients’ socioeconomic status and wellbeing as well as whether patients’ socioeconomic status modified the effect of facilitated access to care. We transformed each of the wellbeing scores to range from 0 (reflecting the worst possible wellbeing) to 100 (reflecting the best possible wellbeing), reversing the PHQ-9, GAD-7, and financial difficulties scale as needed and visualized the disparities in wellbeing by wealth quintiles using an equiplot. We identified dimensions of patient wellbeing that differed significantly by wealth quintiles by comparing a linear regression model with indicator variables for wealth category to an intercept-only model. Next, for each outcome we fitted a linear regression model that included an interaction term between wealth categories and receipt of facilitated access to care and adjusted for the remaining variables in the minimally adjusted model. We used parameters from the model to assess whether a significant association between facilitated access to care and patient wellbeing existed within each wealth category and whether this association was significantly different among wealth categories. We also plotted mean patient wellbeing scores with and without receipt of facilitated access to care by wealth category. Data analysis was performed in Stata/IC version 15.1.

## Results

Of 343 patients contacted for participation in the study, 62% (n = 214) participated in the telephone survey. Non-response was mainly due to not reaching patients on the telephone (86, 25%) or death during the COVID-19 pandemic (41, 12%), with higher non-reachability among non-facilitated patients (n = 74, 30%) compared to the facilitated patients (n = 12, 12%) and equal prevalence of death during the pandemic in both groups (12% vs.12%). Of the 140 patients who were sampled from the EMR and therefore considered to be non-facilitated, 35.7% (n = 50) self-reported having received some types of assistance from PIH/IMB while 9.5% (n = 7) of those who were sampled from the list of beneficiaries receiving facilitated access to care self-reported not receiving any facilitation **(Fig 1**).

Of 214 study participants, the mean age was 50.1 years; 77.6% were female and 22.4% male; 24.8% had no education; 76.2% were rural residents; 23.8% belonged to the poorest *ubudehe* category and 94.4% had membership in community-based health insurance (Table 1). Based on our asset-based wealth index, approximately equal proportions of patients belonged to the wealthiest or second wealthiest quintile of Rwandans as belonged to the bottom three quintiles combined. Slightly less than half of all patients (43.0%) were diagnosed with breast cancer versus all other types of cancer and mean time since cancer diagnosis was 4.4 years. A large majority (95.0%) reported public transportation as their usual transport means to cancer care facility before COVID-19. At the beginning of the lockdown, 64.0% were on chemotherapy treatment, 29.4% were on other treatments and 6.5% had no treatment plan. Compared to patients who did not receive facilitation, patients who received facilitated access to care were significantly more likely to have breast cancer (56.8% vs 35.7%, p<0.01) and were more likely to be on chemotherapy treatment at the start of the pandemic (90.5% vs. 64.0%, p<0.01).

**Table 1:** Baseline characteristics of study participants stratified by receipt of facilitated access to care (N = 214).

Variable name	All participants		No facilitation		Received facilitated access to care		
	N = 214		N = 140		N = 74		
	n	percent	N	Percent	n	Percent	P-value
**Demographic Characteristics**			** **				** **
Age in years, Mean, SD	50.1	±13.3	53	±12.7	49	±14.5	0.17
Gender							
Female	166	77.6%	110	78.6%	56	75.7%	0.63
Male	48	22.4%	30	21.4%	18	24.3%	
Level of education							0.60
None	53	24.8%	36	25.7%	17	23.0%	
Some primary classes	61	28.5%	37	26.4%	24	32.4%	
Primary school graduate	45	21.0%	32	22.9%	13	17.6%	
Some high school classes	30	14.0%	21	15.0%	9	12.2%	
High school graduate	14	6.5%	9	6.4%	5	6.8%	
Bachelor’s degree	11	5.1%	5	3.6%	6	8.1%	
Remoteness of residence ^£^							0.83
Urban	51	23.8%	34	24.3%	17	23.0%	
Rural	163	76.2%	106	75.7%	57	77.0%	
Ubudehe categories (n = 207)							0.82
I	49	23.7%	32	23.3%	17	24.3%	
II	52	42.0%	56	40.9%	31	44.3%	
III	71	34.3%	49	35.8%	22	31.4%	
Insurance type							0.28
No insurance	1	0.5%	0	0.0%	1	1.4%	
Mutuelle / CBHI	202	94.4%	134	95.7%	68	91.9%	
Private insurance	11	5.1%	6	4.3%	5	6.8%	
National Wealth Quintiles							0.12
Quintile 1-3(Poor-Middle)	59	27.6%	40	28.6%	19	25.7%	
Quintile 4(Rich)	76	35.5%	55	39.3%	21	28.4%	
Quintile 5(Richer)	79	36.9%	45	32.1%	34	46.0%	
**Cancer Characteristics**			** **				
Type of cancer							<0.01
Breast cancer	92	43.0%	50	35.7%	42	56.8%	
Non-breast cancer types	122	57.0%	90	64.3%	32	43.2%	
**Duration of diagnosis, in years, Mean, SD**	4.4	±2.4	4.6	±1.3	4.1	±2.7	0.16
**Means of transport to cancer care before COVID-19**							0.37
PIH/IMB’s transport	3	1.4%	1	0.7%	2	2.7%	
Public transport	204	95.3%	133	95.0%	71	96.0%	
Private transport	4	1.9%	3	2.14%	1	1.3%	
By foot	3	1.4%	3	2.14%	0	0.0%	
**Type of treatment at the start of the pandemic**							<0.01
Chemotherapy	137	64.0%	70	50.0%	67	90.5%	
Non-chemo treatments	63	29.4%	60	42.9%	3	4.1%	
No treatment	14	6.6%	10	7.1%	4	5.4%	

During the first 17 months of the COVID-19 pandemic, over half of patients (53.3%) reported missing or delaying a scheduled clinical appointment, 26.6% did not have any in-person oncology encounters and only 9.8% reported any telemedicine consultations (Table 2). About half of patients perceived no changes in the quality of cancer care during the pandemic with approximately equal numbers perceiving care as worsening (23.4%) and as improving (27.1%). One in five (19.2%) cancer patients reported a change of treatment plan due to COVID-19 since the start of the pandemic. The most commonly reported challenges encountered during the pandemic included financial challenges (86.0%), family issues (59.4%), lack of transportation (53.3%), comorbidities (42.1%), logistical challenges related to transport (36.5%), worsened disease (24.8%) and COVID-19 infection among patients or close relatives (12.2%). Compared to patients who did not receive facilitated access to care, patients who received facilitation were significantly more likely to have had at least one in-person clinical encounter during the pandemic (87.8% vs. 64.7%, p<0.01) and to perceive improvements in the quality of cancer care during the pandemic (37.8% vs. 21.4%, p<0.03) and less likely to report access to transportation as a barrier (42.9% vs. 24.3%, p-value<0.01).

**Table 2:** Cancer care during COVID-19 pandemic, stratified by receipt of facilitated access to care (N = 214).

Variable name	All participants		No facilitation		Received facilitated access to care		
	N = 214		N = 140		N = 74		
	Frequency	Percent	Frequency	Percent	Frequency	Percent	P-value
**Patient missed or delayed appointments**							0.20
Yes	114	53.3%	79	56.4%	35	47.3%	
No	100	46.7%	61	43.6%	39	52.7%	
**Number of in-person encounter with oncology clinician**	** **						<0.01
None	57	26.6%	48	34.3%	9	12.2%	
1–2	56	26.2%	49	35.0%	7	9.4%	
3–5	44	20.6%	25	17.9%	19	25.7%	
6–9	31	14.5%	10	7.1%	21	28.4%	
> = 10	26	12.1%	8	5.7%	18	24.3%	
**Had a teleconsultation by an oncology clinician**							0.07
Yes	21	9.8%	10	7.1%	11	14.9%	
No	193	90.2%	130	92.9%	63	85.1%	
**Perceived quality of care during COVID-19 compared to before COVID-19**							0.03
Worse	50	23.4%	36	25.7%	14	18.9%	
About the same	106	49.5%	74	52.9%	32	43.3%	
Better	58	27.1%	30	21.4%	28	37.8%	
**Change of treatment due to COVID-19 pandemic**							0.51
Yes	41	19.2%	25	17.9%	16	21.6%	
No	173	80.8%	115	82.1%	58	78.4%	
**Barriers faced during the pandemic**							
Financial barriers	184	86.0%	86	88.7%	98	83.8%	<0.01
Family issues	127	59.4%	85	60.7%	42	56.8%	0.58
Lack of transportation	114	53.3%	71	50.7%	43	58.1%	0.30
Other comorbidities	90	42.1%	62	44.3%	28	37.8%	0.36
Logistical issues to access transportation *	78	36.5%	60	42.9%	18	24.3%	<0.01
Worsened disease	53	24.8%	36	25.7%	17	23.0%	0.66
COVID-19 infection (patient or relative	26	12.2%	16	11.4%	10	13.5%	0.66

* indicates issues of accessing transport even when it was available. This includes accessing car parking, living far from the pickup points, transport fees… . etc.)

Cancer patients generally scored poorly on wellbeing indicators (Table 3). The quality of life was lowest on the global health status scale (mean = 37.5, SD±22.4) and highest on social functioning scale (mean = 83.0, SD±29.3). For mental health, mean overall PHQ-9 score was 8.4 (SD ±6.7) with 63.1% scoring ≥ 5, a commonly used cut off for mild to severe depression. Mean overall GAD-7 score was 6.4 (SD±5.9), with 55.9% scoring ≥5, a commonly used cut off for mild to severe anxiety. The average COST score was 9.3 SD ±7.9 out of a theoretical maximum of 44 and the average financial difficulties score was 53.1 out of 100. When using two-sample t-tests, only global quality of life was significantly different between patients who did and did not receive facilitate access to care, with quality of life being higher among patients who received facilitated access to care (mean = 42.6, SD±24.6) compared to those who did not (mean-34.8, SD±20.7).

**Table 3:** Patient wellbeing indicators, stratified by receipt of facilitated access to care, N = 214.

	All patients		No facilitation		Facilitated access to care		P-value
			N = 140		N = 74		
	Mean	SD	Mean	SD	Mean	SD	
**Quality of life** ^1^	** **						
Global quality of life	37.5	22.4	34.8	20.7	42.6	24.6	0.02
Physical functioning	60.6	25.4	60.0	23.6	61.6	28.7	0.67
Role functioning	52.9	38.4	49.9	37.5	58.6	39.5	0.12
Emotional functioning	64.1	33.5	64.6	31.9	63.1	36.6	0.74
Cognitive functioning	65.6	32.6	64.6	32.4	67.3	33.0	0.56
Social functioning	83.0	29.3	80.6	30.4	87.6	26.9	0.09
**Mental Health**	** **						** **
PHQ-9^2^	8.4	6.7	8.4	6.4	8.6	7.2	0.87
GAD-7^3^	6.4	5.9	6.3	5.6	6.6	6.4	0.72
**Financial wellbeing**							
COST^4^	9.3	7.9	8.6	7.4	10.7	8.6	0.07
Financial difficulties^5^	53.1	44.7	56.2	44.7	47.3	44.5	0.17

SD: standard deviation from the mean scores

^1^Measured using EORTC-QOL- C30 Version 3, score range from 0 to 100, high scores reflect best quality of life

^2^ Measured using patient health questionnaire (PHQ-9), with scores range from 0–27, higher scores reflect severe degree of depression

^3^ General anxiety measured using GAD-9 questionnaire, with scores ranging from 0–21, higher scores reflects severe degree of anxiety

^4^Financial toxicity measured using COST, which ranges from 0 to 44, higher scores reflect better financial wellbeing

^5^Measured using the EORTC-QOL-C30, scores range from 0 to 100, higher scores represent worse financial wellbeing

These findings were consistent with the findings of our unadjusted regression models, where only global quality of life was significantly associated with facilitated access to care (β = 7.75, 95% CI: 1.5, 14.0, p = 0.01) (Table 4). In the minimally adjusted model, we observed that patients who received facilitated access to care had significantly better outcomes in terms of both global health status scale (β = 9.14, 95% CI: 2.3, 16.0, p <0.01) and improved COST (β = 2.62, 95% CI: 0.2, 5.0, p = 0.03), but no statistically significant changes were observed on depression, anxiety, financial difficulty aspect of quality of life or physical, emotional, role, social or cognitive functioning. These findings persisted in the fully adjusted model. In our sensitivity analysis, using self-reported receipt of facilitated access to care resulted in large attenuation of the effect sizes of the exposure on all outcome indicators with no statistically significant associations (S1 Table). However, when we restricted our analysis to patients whose list-based status matched their self-reported support status, the effect sizes on all outcomes were consistent with the primary analysis results, although the associations no longer reached statistical significance (S2 Table).

**Table 4:** A multivariate linear regression analysis for crude and adjusted beta coefficients for the independent association between receipt of facilitated access to care and patients’ self-reported outcomes.

	Crude model			Minimally Adjusted**				Fully Adjusted***		
	β	95% CI	p-value	β	95% CI		p-value	β	95% CI	p-value
**Quality of life** ^1^										
Global quality of life	7.75	(1.5,14.0)	0.01	9.14	(2.3,16.0)	<0.01		8.60	(1.3,15.8)	0.02
Physical functioning	1.57	(-5.6,8.8)	0.67	1.67	(-6.3,9.6)	0.68		0.45	(-7.8,8.7)	0.91
Role functioning	8.68	(-2.1,19.5)	0.12	10.10	(-1.6,21.7)	0.09		9.36	(-2.6,21.3)	0.12
Emotional functioning	-1.58	(-11.1,7.9)	0.74	-1.20	(-11.6,9.2)	0.82		-2.84	(-13.3,8.21)	0.61
Cognitive functioning	2.70	(-6.5,11.9)	0.56	3.55	(-6.5,13.6)	0.48		3.40	(-7.1,13.9)	0.52
Social functioning	7.02	(-1.2,15.3)	0.09	8.85	(-0.3,18.0)	0.06		6.85	(-2.5,16.2)	0.15
**Mental Health**										
PHQ-9^2^	0.15	(-1.7,2.1)	0.16	-0.31	(-2.4,1.8)	0.77		-0.25	(-2.4,1.9)	0.82
GAD-7^3^	0.31	(-1.3,1.9)	0.72	0.04	(-1.8,1.9)	0.96		0.33	(-1.6,2.2)	0.73
**Financial wellbeing**										
COST^4^	2.05	(-0.2,4.3)	0.07	2.62	(0.2,5.0)	0.03		2.64	(0.2,5.1)	0.03
Financial difficulties^5^	-8.89	(-21.5,3.7)	0.17	-11.42	(-25.2,2.4)	0.10		-10.5	(-25.0,3.8)	0.15

^1^Measured using EORTC-QOL- C30, score range from 0 to 100, higher scores reflect best quality of life

^2^ Measured using patient health questionnaire (PHQ-9), with scores range from 0–27, higher scores reflect severe degree of depression

^3^ General anxiety measured using GAD-9 questionnaire, with scores ranging from 0–21, higher scores reflects severe degree of anxiety

^4^Financial toxicity measured using COST, which ranges from 0 to 44, higher scores reflecting better financial wellbeing.

^5^Measured using the EORTC-QOL-C30, scores range from 0 to 100, higher scores represent worse financial wellbeing

** Minimally adjusted model includes age, wealth quintile, cancer types, duration of cancer diagnosis, treatment type at the beginning of lockdown

***Fully adjusted model included all variables in Table 1

**Fig 3:**
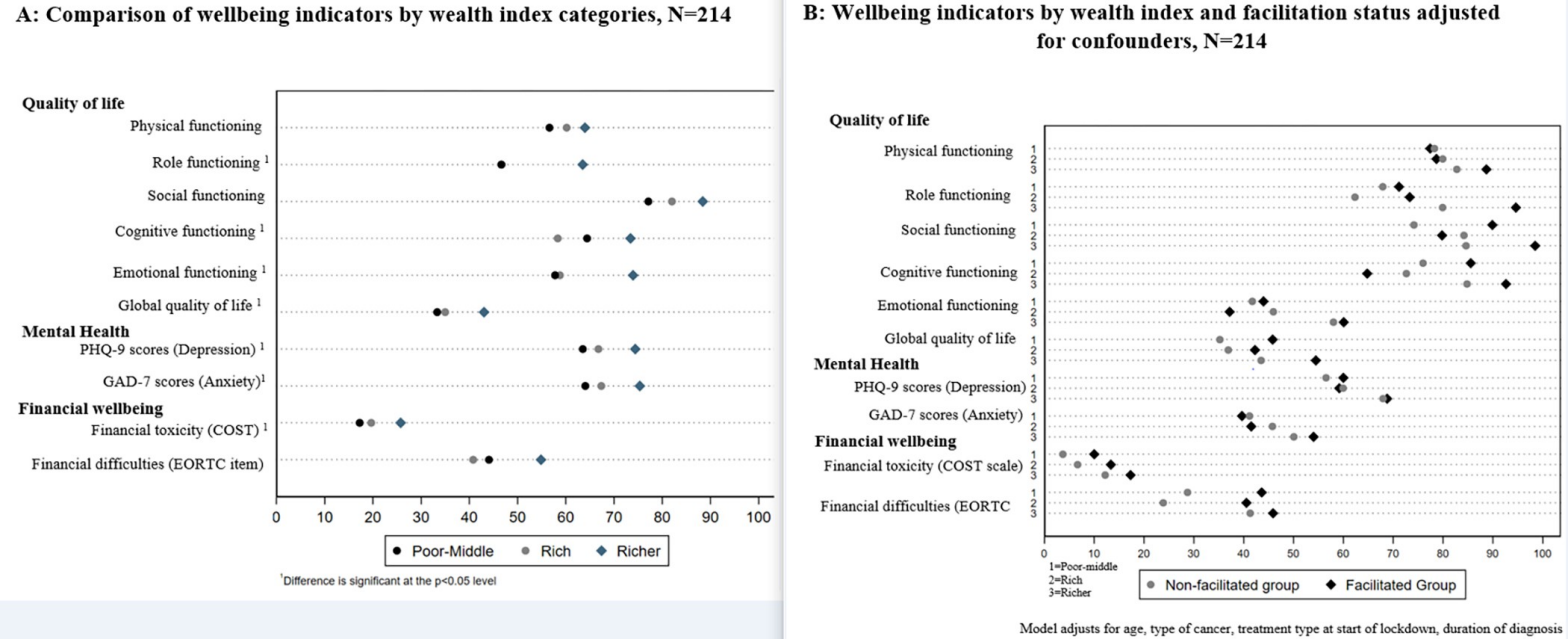
Comparison of wellbeing indicators by wealth index and facilitation status for crude and adjusted models, N = 214.

When assessing patient wellbeing by wealth category, we observed statistically significant differences by wealth category on most wellbeing dimensions, including global quality of life, role functioning, emotional functioning, cognitive functioning, depression, anxiety, and financial toxicity **(Fig 3A).** Poor-middle patients consistently reported worse outcomes than richest patients for all wellbeing outcomes and reported worse outcomes than richer patients for all outcomes except cognitive functioning and financial difficulties. We did not observe evidence of wealth category modifying the association between facilitated access to care and wellbeing indicators; however, facilitation did not lead to equity between the richest and poorest groups and for many outcomes, especially physical functioning, role functioning, emotional functioning, depression, and anxiety, the disparities remain marked **(Fig 3B**).

## Discussion

Our study assessed the association between receipt of facilitated access to care during COVID-19 pandemic and patient wellbeing among cancer patients at Butaro Cancer Center of Excellence (BCCOE), Rwanda. During the pandemic, disruptions in accessing care at BCCOE were widespread, as evidenced by the 53% of respondents who missed or delayed a scheduled appointment and 26% who did not have any in-person contact with their oncology provider during the first 17 months of the COVID-19 pandemic. Despite the remote geographical location of BCCOE and challenges linked to providing cancer care in low income settings, the magnitude of these disruptions are comparable to what was reported in high income settings [39–42]. Furthermore, receipt of facilitated access to care was associated with increased frequency of in-person clinical encounters, perceived improvements in the quality of cancer care, reduced logistical barriers to accessing care, improved global quality of life and reduced financial toxicity among cancer patients. Although not statistically significant, the direction of association between receipt of facilitated access to care and patient wellbeing was also beneficial for the remaining financial wellbeing outcome as well as for four of the five remaining quality of life outcomes. Our findings suggest that providing facilitated access to care during the pandemic was able to mitigate disruptions on access to oncology services during COVID-19 pandemic, particularly during the national lockdowns.

While facilitated access to care was associated with some improvements in quality of life and better financial wellbeing, respondents still exhibited generally low levels of patient wellbeing. Global quality of life scores (mean = 37.5) was lower than the scores reported among hospitalized cancer patients before COVID-19 pandemic in similar LMICs settings (mean = 49.5–54.6) [27, 28]. Similarly, financial toxicity was significantly worse in our study (mean = 9.3) compared to a pre-pandemic study on cancer patients from Nigeria (mean = 26.50) [43]. The prevalence of mild to severe depression (63.1%) and anxiety (55.9%) was also high overall, and receipt of facilitated access to care was not associated with improvements in mental health outcomes. High prevalence of depression and anxiety were reported among cancer patients in Rwanda prior to the pandemic [44, 45], and several studies have reported increased psychological distress among cancer patients during COVID-19 pandemic in developed settings [46, 47]. Collectively, these findings underscore the extreme psychological and economic vulnerability of Rwandan cancer patients during the COVID-19 pandemic.

As has been frequently reported in both high- and low-income countries, our study found statistically significant disparities in patient wellbeing by socioeconomic status, with patients in poor wealth categories reporting worse wellbeing compared to the richest patients [48–53]. While providing facilitated access to cancer care during COVID-19 pandemic benefited patients overall, these services were not sufficient to equalize outcomes by wealth categories. Furthermore, receipt of facilitated access to cancer care was not associated with any markers of baseline socioeconomic status. Instead, clinical factors, such as type of cancer and type of cancer treatment, appeared to dictate which patients received facilitated care support. However, in other settings, COVID-19 has disproportionately worsened poor cancer patients’ abilities to access adequate food, social support, housing insecurity, remote residence, and income [54–56]. These financial hardships during COVID-19 have also been found to correlate with poor mental health and quality of life of cancer patients [57].

While providing all patients with facilitated access to care regardless of socioeconomic status was an appropriate mitigation strategy during COVID-19, poor overall patient wellbeing and disparities by socioeconomic status suggest a need for more robust, equity-minded interventions to promote the wellbeing of cancer patients at BCCOE even after the COVID-19 pandemic. Telemedicine has been one of strategies used to mitigate disruptions to cancer care and to maintain patient-provider interactions during COVID-19 lockdowns; however, in our study, fewer than 10% of patients reported ever having a telehealth session with their health provider during COVID-19 pandemic Improving the feasibility of telehealth would require improving recording and updating of patient’s contact information in electronic medical record (EMR) systems [15]; possibly adding phone numbers of caregivers or local community health worker’s to patients’ EMR file; and providing of telephones to the poorest patients who may not have cell phone in their household. Better access to telecommunications could also promote coordination of transport and medication drop off or provision of psychosocial support. For example, health provider-led telemedicine approach for mental health support among cancer patients was successfully implemented in Mexico during COVID-19 pandemic [58]. While BCCOE currently has a social assistance program to support patients during hospitalization or soon after hospital discharge, creating a holistic nutrition assistance program that ensures patients have enough nutrition throughout their treatment period could promote better patients’ outcomes [59]. Eligibility criteria for these interventions should consider the financial burden posed by the cost of cancer treatment and barriers related to access to care, rather than predetermined social class categories [60].

The present study had several limitations. First, a large portion of patients who were sampled to participate were not reachable or had died. As evidenced by the fact that only a third of our respondents belonged in the bottom 60% of the national wealth distribution, it is likely that our telephone-based data collection excluded a large proportion of the poorest patients. Similarly, patients who were most economically vulnerable or who experienced the greatest disruptions in care may have been more likely to die prior to data collection. Collectively, we would expect these limitations to lead to a respondent population that is better off than the average cancer patient at BCCOE, resulting in an underestimation of poor patient outcomes. Second, we observed differential participation between the facilitated group and non-facilitated group, with the non-facilitated group being less likely to respond. If non-response were associated with worse patient well-being, we would expect this selection bias to underestimate the association between receipt of facilitated access to care and patient wellbeing. Third, facilitated access to care was mainly offered during COVID-19 lockdowns, including the national lockdown of between March 22^nd^ and June 31^st^ 2020 and subsequent small-scale lockdowns, while data collection took place in September 2021. Thus, it is possible that some beneficial effects of facilitation during the first national lockdown had dissipated by the time of data collection. Fourth, some misclassification of exposure to facilitated transport is likely as 26% of sampled patients contradicted their listed facilitation status; however, our sensitivity analysis that included only patients with concordant self-reported and listed exposure status showed results of a similar magnitude as our primary analysis, although the smaller sample size likely reduced our power to detect a significant effect. Furthermore, we would anticipate this misclassification to bias our results towards the null hypotheses of no association between receipt of facilitation and our outcomes of interest. Fifth, all data was self-reported and collected via telephone. Since the study was conducted by PIH/IMB, which is known to provide socioeconomic assistance to cancer patients, patients may have been motivated to over-emphasize hardships in effort to gain further assistance. Telephone surveys may be more vulnerable to mis-reporting than in-person surveys; however, given the increased risks of COVID-19 among cancer patients, we did not feel that in-person interviews would be ethical at the time of data collection. Sixth, while the EORTC QLQ-C30, PHQ-9, GAD-7, and COST-FACIT tools have been validated in similar settings including neighboring countries of Rwanda [36, 43, 44, 61–64], the validity and reliability of these tools among cancer population in Rwanda was not assessed to account for cultural variations in this study. Lastly, residual confounding is possible as unmeasured factors, including quality of the clinician-patient relationship, patient health-related knowledge, phases of cancer treatment, or patients’ social capital may be associated both being receiving facilitated access to care and subsequent patient wellbeing outcomes.

## Conclusion

Provision of facilitated transportations during COVID-19 pandemic was associated with more frequent in-person clinical encounter, better perceived care, greater global quality of life, and lower financial toxicity among patients receiving cancer care in rural Rwanda. However, cancer patients still reported disruptions to care and reported low overall levels of wellbeing, with socioeconomic disparities in patient wellbeing persisting despite the provision of facilitated transport to continue access to oncology care. Preemptively implementing stronger and equity-minded care facilitation and better patient outreach programs may promote better care, strengthen patient outcomes, and leave cancer centers better prepared to respond to future crises.

## Supporting information

S1 Table A multivariate linear regression analysis for crude and adjusted beta coefficients for the independent association between self-reported receipt of facilitated access to care during COVID-19 pandemic and patient self-reported outcomes (Facilitated group n = 117; Non-facilitated group n = 97). (DOCX)

S2 Table A multivariate linear regression analysis for crude and adjusted beta coefficients for the independent association between concordant report of receipt of facilitated access to care during COVID-19 pandemic and patient self-reported outcomes (Facilitated group n = 67; Non-facilitated group n = 90). (DOCX)

S1 Data STATA dataset and do file. (ZIP)
