# Supplementary material for: Impact of facilitating continued accessibility to cancer care during COVID-19 lockdown on perceived wellbeing of cancer patients at a rural cancer center in Rwanda
Source: PLOS Glob Public Health. 2023 Feb 27;3(2):e0001534. doi: 10.1371/journal.pgph.0001534 (PMC10021873; doi:10.1371/journal.pgph.0001534)
Supplement: S1 Table — (DOCX) [file pgph.0001534.s001.docx]

**S1 Table:** A multivariate linear regression analysis for crude and adjusted beta coefficients for the independent association between self-reported receipt of facilitated access to care during COVID-19 pandemic and patient self-reported outcomes (Facilitated group n=117; Non-facilitated group n=97)

| **Wellbeing outcomes** | **Crude model** | | | **Minimally Adjusted**** | | | | | | | | **Fully Adjusted***** | | | | | |
| --- | --- | --- | --- | --- | --- | --- | --- | --- | --- | --- | --- | --- | --- | --- | --- | --- | --- |
|  | **β** | **95% CI** | **p-value** | **β** | | **95% CI** | | | | **p-value** | | **β** | | **95% CI** | | **p-value** | |
| **Quality of life ^1^** |  |  |  |  | |  | | | |  | |  | |  | |  | |
| General quality of life | 1.81 | (-4.2,7.9) | 0.56 | 3.60 | | (-3.5,10.7) | | | | 0.32 | | 3.16 | | (-4.5,10.8) | | 0.42 | |
| Physical function | -1.43 | (-8.3,5.4) | 0.68 | 0.49 | | (-7.6,8.6) | | | | 0.90 | | -2.11 | | (-10.8,6.6) | | 0.63 | |
| Role functioning | -4.47 | (-14.8,5.9) | 0.4 | -0.66 | | (-12.7,11.3) | | | | 0.91 | | -1.38 | | (-14.1,11.3) | | 0.83 | |
| Emotional function | -2.18 | (-11.3,6.9) | 0.63 | 1.07 | | (-9.6,11.7) | | | | 0.84 | | -0.01 | | (-11.6,11.6) | | 0.99 | |
| Cognitive function | -6.71 | (-15.5,2.1) | 0.13 | -4.40 | | (-14.7,5.9) | | | | 0.41 | | -2.26 | | (-13.4,8.9) | | 0.69 | |
| Social function | -1.20 | (-9.1,6.7) | 0.77 | 0.45 | | (-9.0,9.9) | | | | 0.92 | | 0.91 | | (-9.0,10.9) | | 0.86 | |
| **Mental Health** |  |  |  |  | |  | | | |  | |  | |  | |  | |
| PHQ-9 scores ^2^ | 0.90 | (-0.9,2.7) | 0.33 | 0.27 | | (-1.8,2.4) | | | | 0.80 | | 0.34 | | (-2.0,2.6) | | 0.77 | |
| GAD-7 Scores ^3^ | 0.79 | (-0.8,2.4) | 0.32 | 0.70 | | (-1.2,2.6) | | | | 0.46 | | 0.90 | | (-1.1,2.9) | | 0.37 | |
| **Financial Wellbeing** |  |  |  |  | |  | | | |  | |  | |  | |  | |
| COST ^4^ | -0.8 | (-2.9,1.3) | 0.46 | -0.45 | | (-2.9,2.1) | | | | 0.72 | | -0.67 | | (-3.3,1.9) | | 0.61 | |
| Financial difficulties ^5^ | -0.27 | (-12.4,11.9) | 0.96 | -0.39 | | (-14.6,13.8) | | | | 0.96 | | -0.52 | | (-15.8,14.8) | | 0.95 | |
|  | | |  |  | |  | |  | |  | |  | |  | | |  |
| ^1^Measured using EORTC-QOL- C30, score range from 0 to 100, high scores reflect best quality of life  ^2^ Measured using patient health questionnaire (PHQ-9), with scores range from 0-27, high scores reflect severe degree of depression  ^3^ General anxiety measured using GAD-9 questionnaire, with scores ranging from 0-21, high score reflects severe degree of anxiety  ^4^Financial toxicity measured using COST, which ranges from 0 to 44, high scores reflecting better financial wellbeing.  ^5^Measured using the EORTC-QOL-C30, scores range from 0 to 100, high scores represent worse financial wellbeing | | | | | | | | | | | | | | | | | |
| ^**^ Minimally adjusted model includes age, wealth quintile, cancer types, duration of cancer diagnosis, treatment type at the beginning of lockdown | | | | | | | | | | | | | | | | | |
| ^***^Fully adjusted model included all variables in table 1 | | | | |  | |  | |  | |  | |  | |  | | |
