# Supplementary material for: Impact of facilitating continued accessibility to cancer care during COVID-19 lockdown on perceived wellbeing of cancer patients at a rural cancer center in Rwanda
Source: PLOS Glob Public Health. 2023 Feb 27;3(2):e0001534. doi: 10.1371/journal.pgph.0001534 (PMC10021873; doi:10.1371/journal.pgph.0001534)
Supplement: S2 Table — (DOCX) [file pgph.0001534.s002.docx]

**S2 Table:** A multivariate linear regression analysis for crude and adjusted beta coefficients for the independent association between concordant report of receipt of facilitated access to care during COVID-19 pandemic and patient self-reported outcomes (Facilitated group n=67; Non-facilitated group n=90)

| **Wellbeing outcomes** | **Crude model** | | | **Minimally Adjusted**** | | | **Fully Adjusted***** | | |
| --- | --- | --- | --- | --- | --- | --- | --- | --- | --- |
|  | **β** | **95% CI** | **p-value** | **β** | **95% CI** | **p-value** | **β** | **95% CI** | **p-value** |
| **Quality of life ^1^** |  |  |  |  |  |  |  |  |  |
| General quality of life | 6.58 | -0.8,13.9) | 0.08 | 8.90 | (-0.42,18.2) | 0.08 | 8.04 | (-2.2,18.3) | 0.12 |
| Physical function | 0.26 | (-8.1, 8.7) | 0.95 | 2.64 | (-7.9,13.2) | 0.62 | 0.62 | (-12.0,10.8) | 0.91 |
| Role functioning | 3.14 | (-9.2,15.5) | 0.62 | 8.89 | (-6.5,24.3) | 0.25 | 4.77 | (-11.5,21.0) | 0.56 |
| Emotional function | -2.78 | (-13.5, 7.9) | 0.61 | -3.66 | (-17.4,10.1) | 0.60 | -5.34 | (-20.3,9.7) | 0.48 |
| Cognitive function | -2.29 | (-12.7, 8.1) | 0.66 | -0.62 | (-13.0,12.6) | 0.92 | -1.46 | (-15.7,12.8) | 0.84 |
| Social function | 4.23 | (-4.6, 13.1) | 0.35 | 7.64 | (-3.7,19.0) | 0.18 | 7.65 | (-4.1,19.4) | 0.20 |
| **Mental Health** |  |  |  |  |  |  |  |  |  |
| PHQ-9 scores ^2^ | 0.64 | (-1.6, 2.8) | 0.57 | 0.57 | (-2.2,3.4) | 0.69 | 0.66 | (-2.4,3.7) | 0.67 |
| GAD-7 Scores ^3^ | 0.70 | (-1.1,2.5) | 0.45 | 1.23 | (-1.1,3.6) | 0.31 | 1.13 | (-1.2,3.9) | 0.29 |
| **Financial Wellbeing** |  |  |  |  |  |  |  |  |  |
| COST ^4^ | 0.88 | (-1.6, 3.4) | 0.49 | 1.96 | (-1.2,5.1) | 0.22 | 1.65 | (-1.8,5.1) | 0.35 |
| Financial difficulties ^5^ | -6.31 | (-20.4,7.8) | 0.39 | -8.89 | (-26.5,8.6) | 0.32 | -8.71 | (-28.6,9.2) | 0.31 |
| ^1^Measured using EORTC-QOL- C30, score range from 0 to 100, high scores reflect best quality of life  ^2^ Measured using patient health questionnaire (PHQ-9), with scores range from 0-27, high scores reflect severe degree of depression  ^3^ General anxiety measured using GAD-9 questionnaire, with scores ranging from 0-21, high score reflects severe degree of anxiety  ^4^ Financial toxicity measured using COST, which ranges from 0 to 44, high scores reflecting better financial wellbeing.  ^5^ Measured using the EORTC-QOL-C30, scores range from 0 to 100, high scores represent worse financial wellbeing  ** Minimally adjusted model includes age, wealth quintile, cancer types, duration of cancer diagnosis, treatment type at the beginning of lockdown  ***Fully adjusted model included all variables in table 1 | | | | | | | | | |
|  | | | | | | | | |  |
